# Intraocular delivery of ZIF-90-RhB-GW2580 nanoparticles prevents the progression of photoreceptor degeneration

**DOI:** 10.1186/s12951-023-01794-6

**Published:** 2023-02-06

**Authors:** Peipei Cao, Yue Cheng, Zhi Li, Ya-Jia Cheng, Xiaoqi Chu, Chao Geng, Xuebo Yin, Yuhao Li

**Affiliations:** 1 Medical International Collaborative Innovation Center, School of Medicine, Nankai University, Tianjin, 300071 China; 2 Beijing Municipal Geriatric Medical Research Center, Xuanwu Hospital, National Neurological Disease Center, Capital Medical University, Beijing, 100053 China; 3 Department of Chemistry, School of Science, Tianjin University, Tianjin, 300072 China; 4 Tianjin Key Laboratory of Food Science and Health, School of Medicine, Nankai University, Tianjin, 300071 China; 5 College of Chemistry and Chemical Engineering, Shanghai University of Engineering Science, Shanghai, 201620 China

**Keywords:** GW2580, ZIF-90, Photoreceptor, Degeneration, Microglia, Intraocular delivery

## Abstract

Photoreceptor degeneration is one of the major causes of progressive blindness which lacks of curative treatment. GW2580, a highly selective inhibitor of colony-stimulating factor 1 receptor, has the protective potential on neurons; however, little was known about the application of GW2580 on photoreceptor degeneration. In this study, BV-2 and 661W cells coculture system was constructed to investigate the interaction between microglia and photoreceptors. GW2580 was loaded into zeolitic imidazolate framework-90-rhodamine B (ZIF-90-RhB) to synthesize a novel kind of nanoparticles, namely, ZIF-90-RhB-GW2580, through a one-step self-assembly approach. A photoreceptor degeneration model was generated by intense light exposure in zebrafish and ZIF-90-RhB-GW2580 nanoparticles were delivered by the intraocular injection. The results showed that in vitro GW2580 treatment promoted phenotypic transformation in microglia and led to the blockade of photoreceptor apoptosis. Following the intraocular delivery of ZIF-90-RhB-GW2580 nanoparticles, the microglial proliferation and inflammatory response were significantly inhibited; moreover, the photoreceptors underwent alleviated injury with a recovery of retinal structure and visual function. In conclusion, the intraocular injection of ZIF-90-RhB-GW2580 at the early stage enables the precise delivery and sustained release of the GW2580, thus preventing the progression of photoreceptor degeneration.

**Graphical Abstract:**

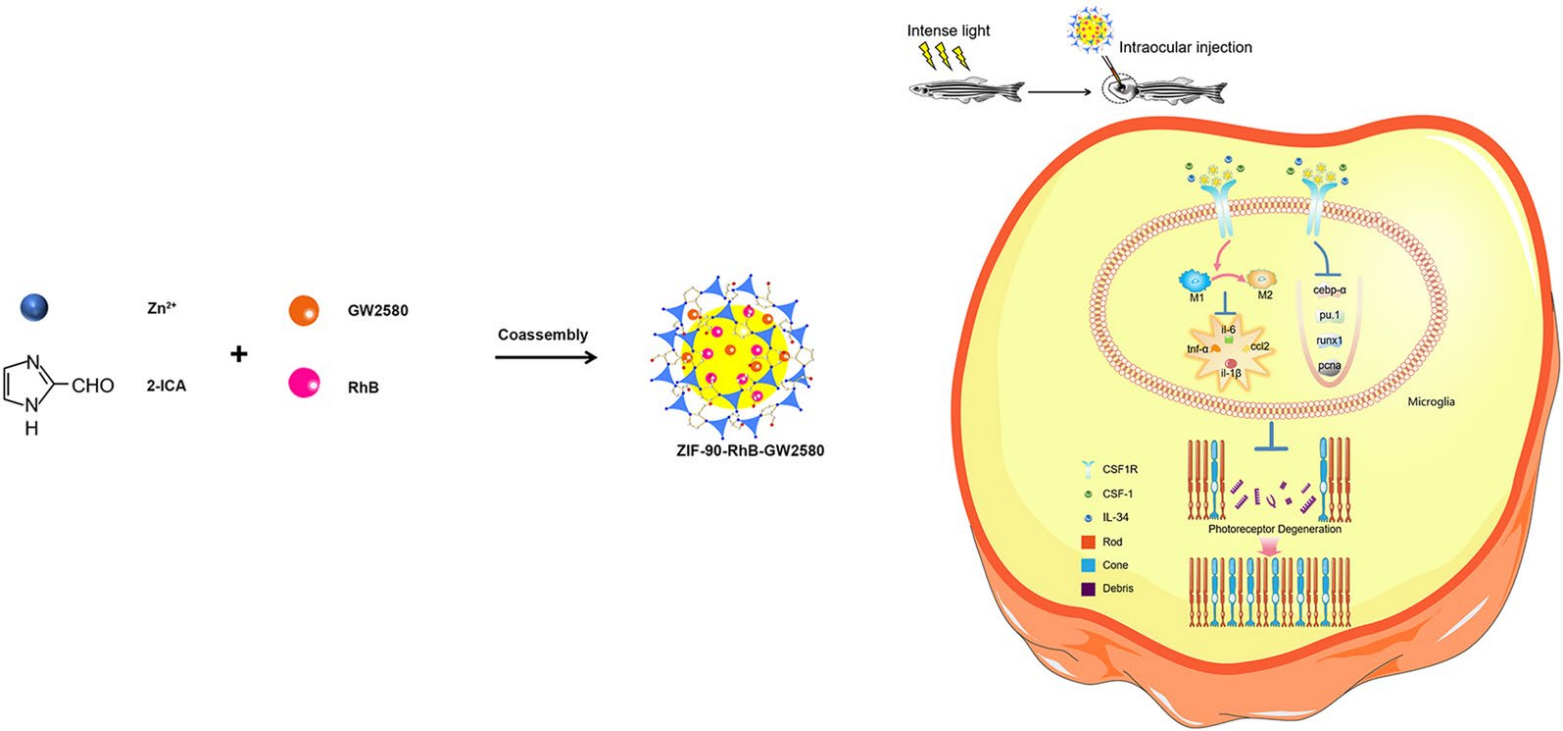

**Supplementary Information:**

The online version contains supplementary material available at 10.1186/s12951-023-01794-6.

## Background

Photoreceptor degeneration is one of the leading causes of impaired vision and blindness. In many human ocular diseases, such as retinitis pigmentosa, age-related macular degeneration, diabetic retinopathy and glaucoma, the irreversible death of photoreceptors (including cones and rods) is the principal event of photoreceptor degeneration, which lacks of curative treatment [1]. In recent years, zebrafish (*Danio rerio*) have become an ideal animal model of retinal degenerative diseases. On the one hand, the gene expression patterns, structure, function and circadian rhythm of the zebrafish retina are very similar to those of the human retina [2]. On the other hand, there is a notable difference in neuronal regeneration between zebrafish and humans. Unlike the poor self-repair capacity in the human retina, any insult that significantly depletes retinal neurons can trigger robust regeneration in zebrafish, providing a feasible tool for exploring the therapeutic response to potential medications [3].

Microglia are the main resident immune response cells in the retina and monitor the microenvironment continuously [4]. In response to retinal injury, microglia are recruited, activated and proliferated, accompanied by the secretion of proinflammatory cytokines, chemokines and reactive oxygen species, which are closely related to the progression of photoreceptor degeneration [5, 6]. Colony-stimulating factor 1 receptor (CSF1R), a tyrosine kinase receptor, regulates the proliferation, differentiation and survival of mononuclear phagocytes, including microglia, by binding to its cognate ligands, colony-stimulating factor-1 (CSF-1) and interleukin-34 (IL-34) [7]. GW2580 is a highly selective CSF1R inhibitor. Recently, it was reported that GW2580 reduced the number of microglia and suppressed neuroinflammation in animal models of several neurodegenerative diseases [8–10]. However, there is still a lack of knowledge about the effect of GW2580 on photoreceptor degeneration.

Nanoparticles are composed of various biodegradable materials, such as polymers, lipids, phospholipids and even metals. The encapsulation of drugs in nanoparticles can protect drugs from being rapidly degraded, maintain the release period of the drug, and penetrate the physiological barriers of sclera or retina in the cases of intravitreal or periocular injection [11]. Liposomes, nanospheres, dendrimers, hydrogels, and nanoemulsions were used as the nanocarriers in the treatment of different vitreoretinal diseases, such as retinopathy, retinal degeneration, and uveitis [12]. Zeolitic imidazole frameworks (ZIFs) are a class of metal organic frameworks formed by the self-assembly of Zn^2+^ coordination and imidazole ligands. Recently, ZIFs have gained more and more attention as the drug carriers due to their low toxicity, intrinsic biodegradability, easy encapsulation, high drug loading and controlled release [13–15]. Zn^2+^ is a trace element in the human body, and imidazole is a component of amino acids that can be utilized by the body [16, 17]. As a member of ZIFs, ZIF-90 features a large surface area and has great thermochemical stability and tunable porosity; besides, the large channels are suitable for carrying polycyclic drugs [18–20]. Moreover, ZIF-90 may emit fluorescence by tuning the encapsulation and release of fluorochrome, e.g. rhodamine B (RhB), providing a probe for imaging in live cells [21]. Until now, ZIF-90 was mostly used in the delivery of anticancer drugs to tumor tissues [22]; little was known about the application of ZIF-90 as an intraocular carrier.

The aim of this study was to exploit the potential application of GW2580 in the treatment of photoreceptor degeneration. First, we examined the status of microglia and apoptosis of photoreceptor following GW2580 treatment using cell models. Second, we synthesized nanoscale ZIF-90-rhodamine B (ZIF-90-RhB) and evaluated its imaging capability and biotoxicity. ZIF-90-RhB was then used as the carrier skeleton and GW2580 was assembled to prepare a novel kind of nanoparticles, namely, ZIF-90-RhB-GW2580. Third, we investigated the microglia-mediated inflammation and the morphological and functional changes of retina following intraocular delivery of ZIF-90-RhB-GW2580 nanoparticles at the early stage of photoreceptor degeneration in a zebrafish model. The present work not only reveals the therapeutic effect of GW2580 on photoreceptor degeneration but also opens an avenue for the intraocular delivery of ZIFs.

## Results

### Phenotypic transformation and inflammatory response in microglia

**Fig. 1:**
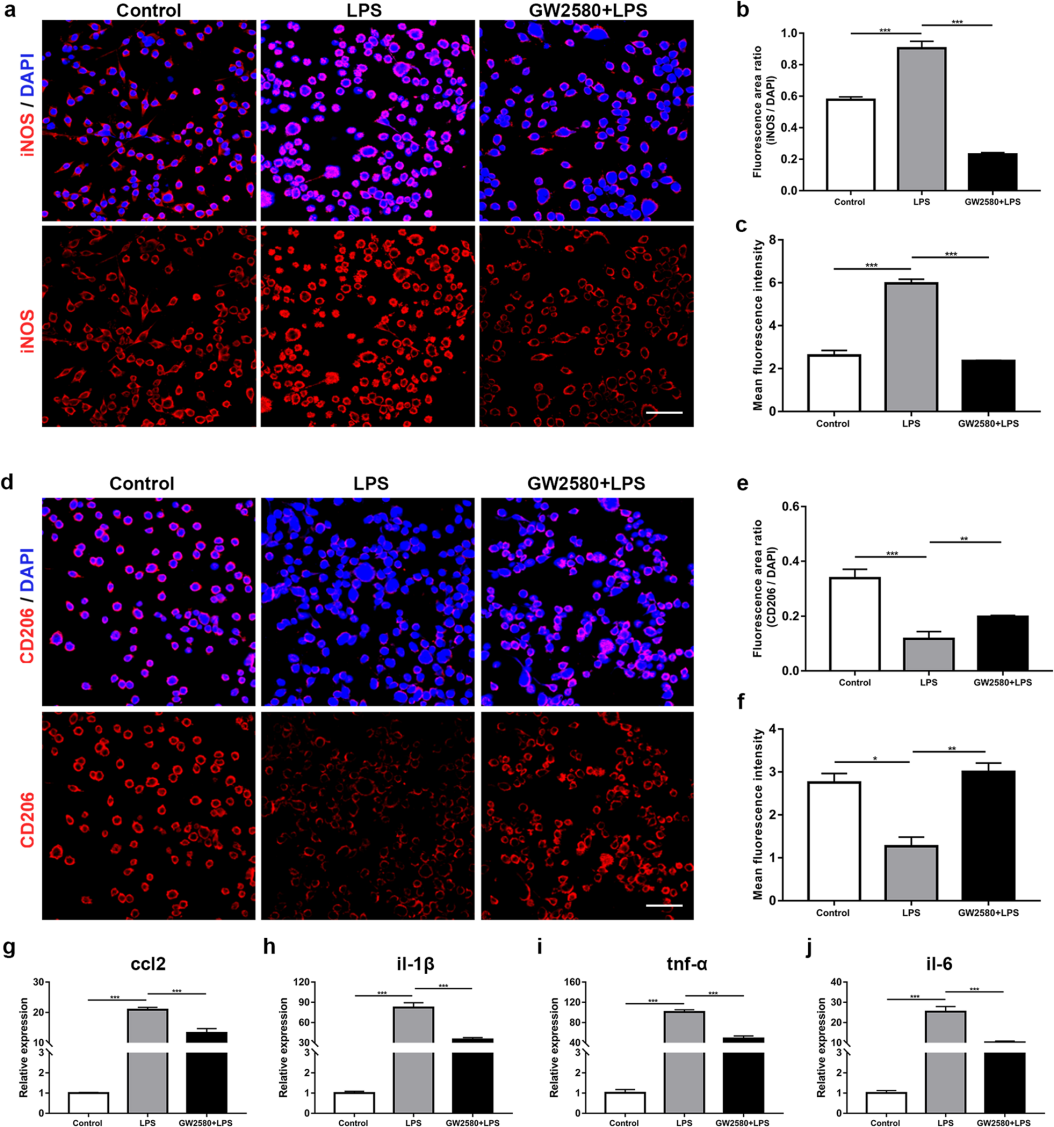
The expression of iNOS, CD206 and proinflammatory factors in BV-2 cells. **a** Images of iNOS immunofluorescence staining in unstimulated (control group), LPS-stimulated (LPS group) and GW2580-treated (GW2580 + LPS group) BV-2 cells. **b**, **c** Quantification of **b** the ratio of iNOS/DAPI area and **c** the mean intensity of iNOS-expressing cells (ANOVA; ***P < 0.001). **d** Images of CD206 immunofluorescence staining in BV-2 cells from the three groups. **e**, **f** Quantification of **e** the ratio of CD206/DAPI area and **f** the mean intensity of CD206-expressing cells (ANOVA; *P < 0.05, **P < 0.01, ***P < 0.001). **g**–**j** The relative expression levels of the proinflammatory factors ccl2, il-1β, tnf-α and il-6 mRNA in BV-2 cells from the three groups (ANOVA; ***P < 0.001). Scale bars in **a** and **d** 20 μm

Following lipopolysaccharide (LPS) stimulation, the expression levels of csf1r, csf-1 and il-34 were significantly increased and peaked at 24 h (Additional file 1: Fig. S1a-c; ANOVA, ***P < 0.001), indicating that CSF1R and its ligands were elevated in the activated microglia. iNOS and CD206 were markers of microglia in M1 and M2 phenotypes, respectively [23]. LPS stimulation significantly led to the high expression of iNOS and the low expression of CD206; however, when BV-2 cells were treated with GW2580 before LPS stimulation, the expression of iNOS was significantly decreased, while that of CD206 was increased, in terms of both fluorescence area and intensity (Fig. 1a–f; ANOVA, *P < 0.05, **P < 0.01, ***P < 0.01). Meanwhile, the expression levels of inflammatory factors ccl2, il-1β, tnf-α and il-6 were increased markedly in the LPS group; however, they were significantly reduced in the GW2580 + LPS group (Fig. 1g–j; ANOVA, ***P < 0.001). These data reveal that GW2580 promotes the phenotypic transformation of microglia from M1 to M2 and alleviates the inflammatory response.

### Photoreceptor apoptosis following GW2580 treatment

**Fig. 2:**
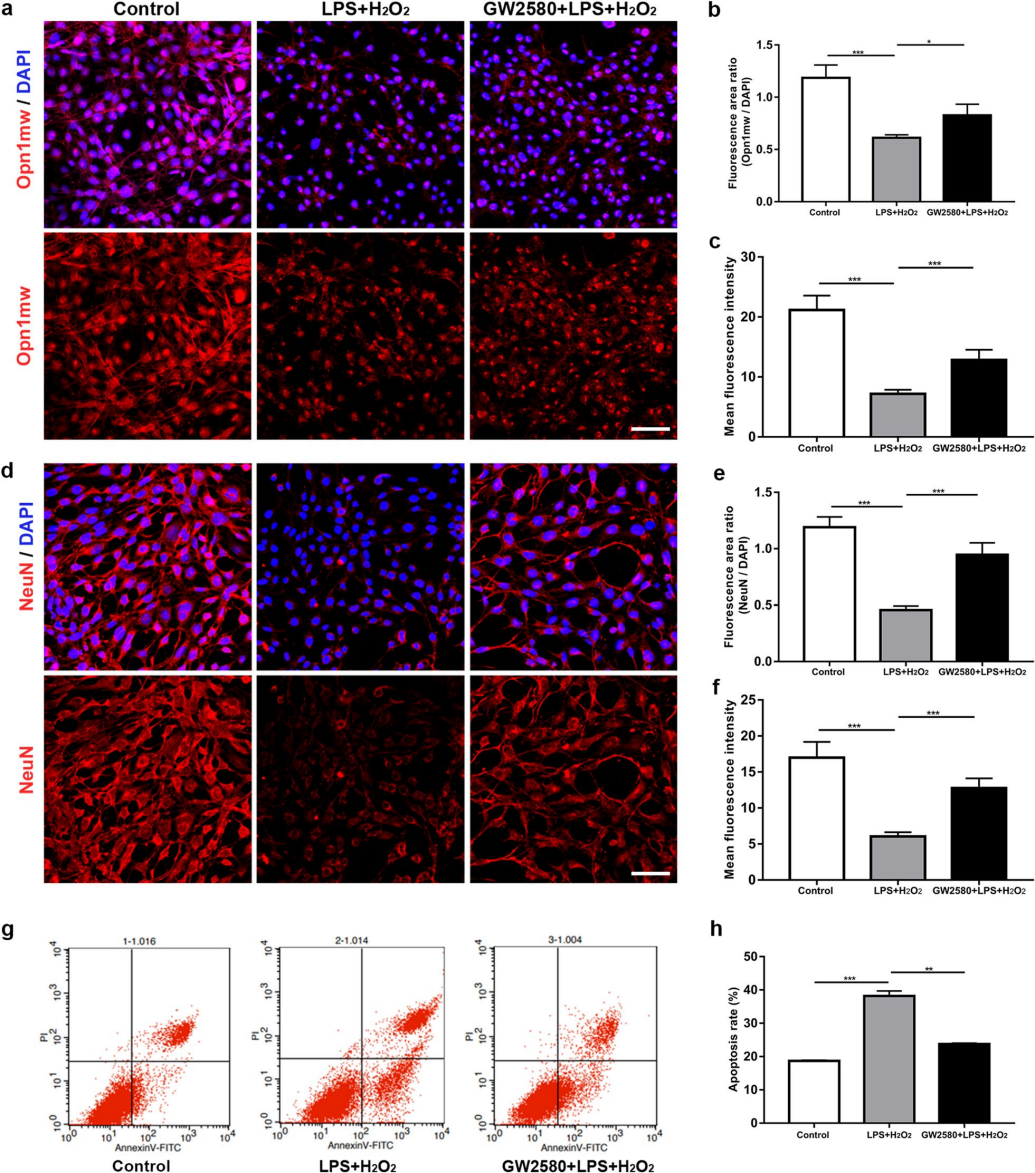
The expression of Opn1mw and NeuN and the apoptosis in 661W cells. **a** Images of Opn1mw immunofluorescence staining in uninduced 661W cells (control group), H_2_O_2_-induced 661W cells cocultured with BV-2 cells following LPS stimulation (LPS + H_2_O_2_ group) and H_2_O_2_-induced 661W cells cocultured with BV-2 cells following GW2580 treatment and LPS stimulation (GW2580 + LPS + H_2_O_2_ group). **b**, **c** Statistical analysis of **b** the ratio of Opn1mw/DAPI area and **c** the mean intensity of Opn1mw-expressing cells (ANOVA; *P < 0.05, ***P < 0.001). **d** Images of NeuN immunofluorescence staining in 661W cells from the three groups. **e**, **f** Statistical analysis of **e** the ratio of NeuN/DAPI area and **f** the mean intensity of NeuN-expressing cells (ANOVA; ***P < 0.001). **g** Flow cytometry of Annexin V-FITC and propidium iodide (PI) staining in 661W cells from the three groups. **h** Quantitative analysis of the apoptosis rate (ANOVA; **P < 0.01, ***P < 0.001). Scale bars in **a** and **d**: 20 μm

The mouse photoreceptor cell line 661W was often used to investigate the molecular basis of retinal diseases as well as therapeutic interventions, since it was able to differentiate into neuronal cells and combined the characteristics of retinal ganglion cells and photoreceptor cells [24]. In this study, BV-2 and 661W cells coculture system was constructed to investigate the interaction between microglia and photoreceptors. The expression profiles of the cone marker Opn1mw and the neuron marker NeuN were detected to evaluate the functional characteristics of photoreceptor. In the LPS + H_2_O_2_ group, the number of Opn1mw- or NeuN-positive cells was obviously reduced; however, GW2580 treatment resulted in significant increases in the Opn1mw- and NeuN-expressing area ratio and intensity (Fig. 2a–f; ANOVA, *P < 0.05, ***P < 0.001). Annexin V-FITC/PI staining was used to manifest the early and late stages of photoreceptor apoptosis, respectively. It was observed that ~ 19% of the photoreceptors in the control group underwent apoptosis, while the percentage was ~ 38% in the LPS + H_2_O_2_ group. With GW2580 treatment, the apoptosis rate was significantly reduced to ~ 24% in the GW2580 + LPS + H_2_O_2_ group (Fig. 2g, h; ANOVA, **P < 0.01, ***P < 0.001). Taken together, the alleviation of microglia-mediated inflammatory response by GW2580 treatment leads to a blockade of photoreceptor apoptosis.

### Characterization of ZIF-90-RhB and ZIF-90-RhB-GW2580

**Fig. 3:**
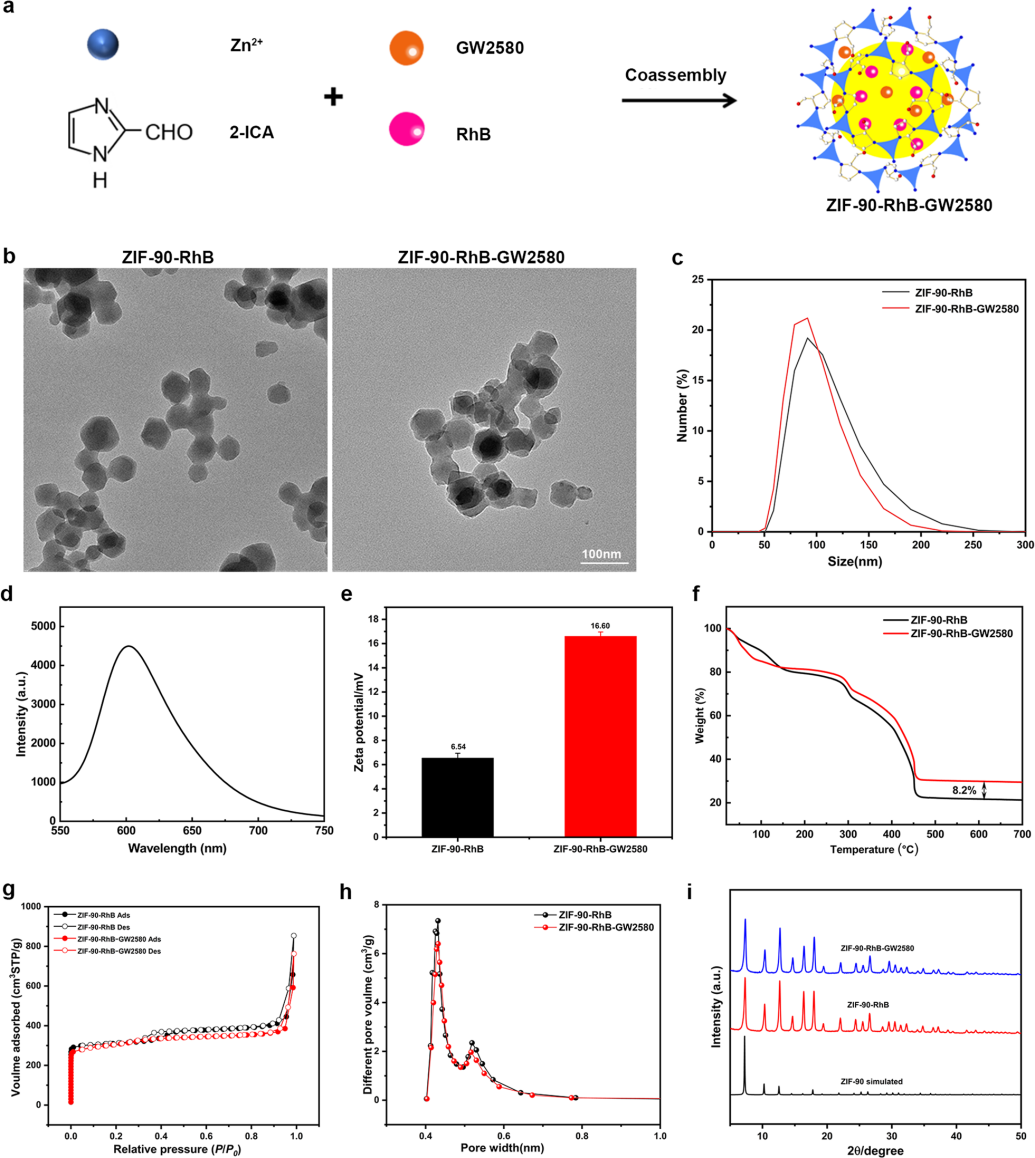
Characterization of ZIF-90-RhB and ZIF-90-RhB-GW2580. **a** Scheme for the synthesis of ZIF-90-RhB-GW2580 nanoparticles. **b** TEM images of ZIF-90-RhB and ZIF-90-RhB-GW2580. **c** DLS of ZIF-90-RhB and ZIF-90-RhB-GW2580 in PBS solution. **d** Fluorescence spectra of ZIF-90-RhB-GW2580. **e** Zeta potentials of ZIF-90-RhB (black) and ZIF-90-RhB-GW2580 (red). **f** Thermogravimetric analysis (TGA) of ZIF-90-RhB (black) and ZIF-90-RhB-GW2580 (red). **g** Adsorption and desorption isotherms of ZIF-90-RhB (black) and ZIF-90-RhB-GW2580 (red). **h** Pore size distributions of ZIF-90-RhB (black) and ZIF-90-RhB-GW2580 (red). **i** X-ray diffraction patterns of ZIF-90-simulated (black), ZIF-90-RhB (red) and ZIF-90-RhB-GW2580 (blue). Scale bar in **a**: 100 nm

A schematic illustration of ZIF-90-RhB-GW2580 is shown in Fig. 3a. Transmission electron microscopy (TEM) images showed that the average sizes of ZIF-90-RhB and ZIF-90-RhB-GW2580 were 55 and 60 nm, respectively (Fig. 3b). Dynamic light scattering (DLS) confirmed that the hydration particle sizes of ZIF-90-RhB and ZIF-90-RhB-GW2580 were about 90 nm (Fig. 3c). The fluorescence emission peak of ZIF-90-RhB-GW2580 was at 602 nm, indicating the successful surface modification of imaging agent rhodamine B (Fig. 3d). The zeta potential of ZIF-90-RhB was 6.54 mV, while the encapsulating of GW2580 increased the positive charge to 16.60 mV, attributed to the -NH_2_ group on GW2580 (Fig. 3e). The Fourier transform infrared (FTIR) spectrum of ZIF-90-RhB-GW2580 showed a peak at 3150 cm^−1^ which was ascribed to the characteristic absorption of the benzene ring in the GW2580 (Additional file 2: Fig. S2). The composition and thermal stability were detected by thermogravimetric analysis (TGA). The weight loss occurred at approximately 450 °C due to the collapse of the framework, indicating the good thermal stability of ZIF-90-RhB and ZIF-90-RhB-GW2580. The drug loading of GW2580 was calculated to be ∼8.2 wt% by comparing the respective weight loss data (Fig. 3f). The N_2_ adsorption–desorption isotherms of ZIF-90-RhB and ZIF-90-RhB-GW2580 were classified as type II, indicating the presence of mesopores [25]. The saturated uptakes for ZIF-90-RhB and ZIF-90-RhB-GW2580 were 853.88 and 762.58 cm^3^g^−1^ (Fig. 3g), while the Brunauer–Emmett–Teller (BET) surface areas were 1246 and 1164 m^2^g^−1^, respectively. The pore volumes of ZIF-90-RhB and ZIF-90-RhB-GW2580 were 7.34 and 6.40 cm^3^ g^−1^, respectively (Fig. 3h). The results demonstrated that the porous framework of ZIF-90-RhB-GW2580 still existed except for a slight decrease in BET due to the successful loading and occupation with GW2580. The powder X-ray diffraction (PXRD) was used to confirm the identity and phase purity. As shown in Fig. 3i, ZIF-90-RhB nanocrystals (red) had similar peaks to the simulated ones of pure ZIF-90 crystals (black). In addition, we observed that the X-ray patterns of ZIF-90-RhB and ZIF-90-RhB-GW2580 were in good agreement, as well as the minimal effect of GW2580 encapsulation on lattice distortion of ZIF-90-RhB, clearly indicating that the pure phase ZIF-90-RhB-GW2580 was obtained. The above data indicate that ZIF-90-RhB and ZIF-90-RhB-GW2580 are successfully synthesized.

### Bioimaging capability of ZIF-90-RhB

**Fig. 4:**
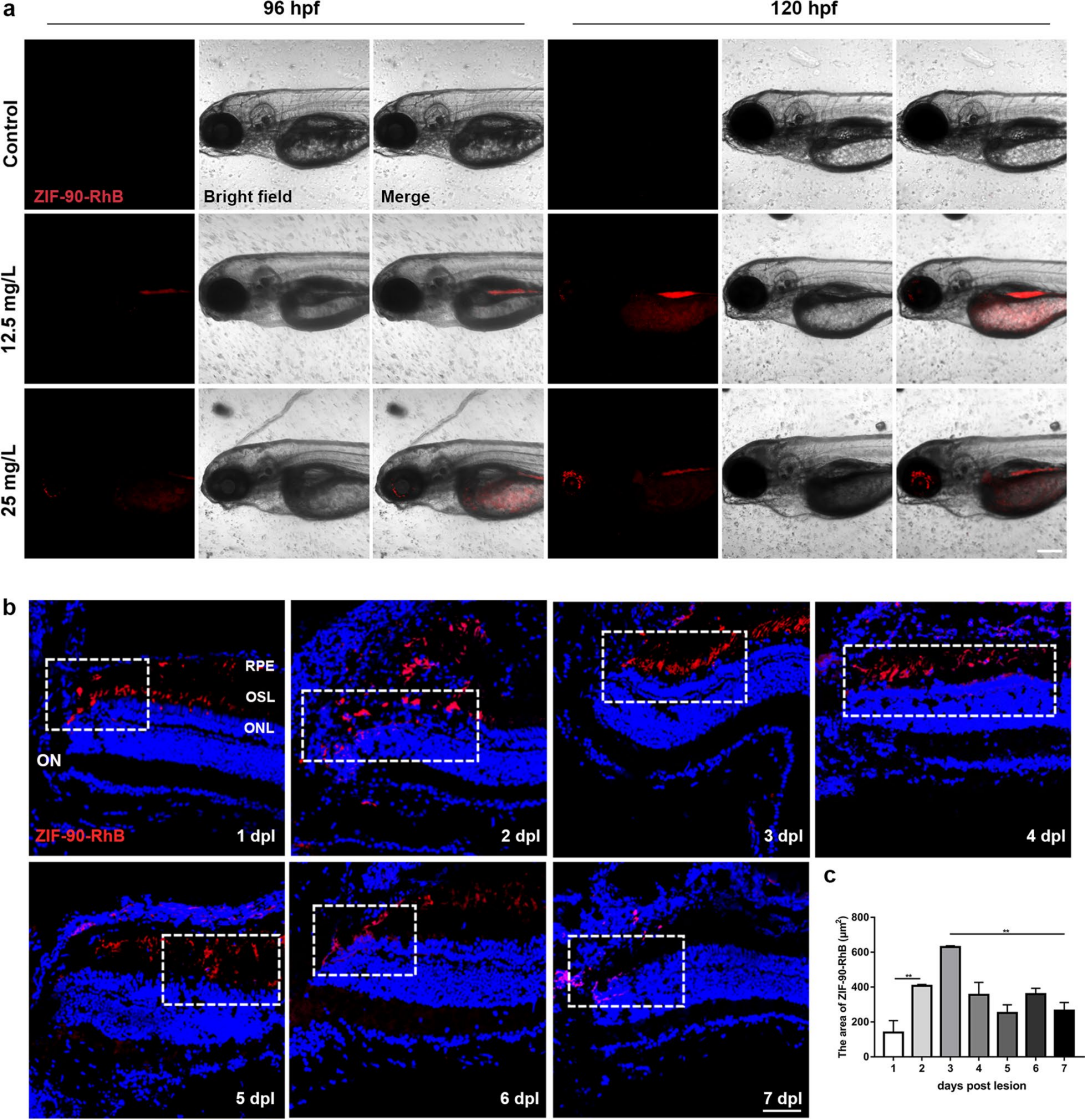
The distribution of ZIF-90-RhB in larval zebrafish and light-lesioned retina. **a** Bright field and fluorescence images of larval zebrafish following ZIF-90-RhB exposure at 96 and 120 hpf. **b** Time-lapse localization of ZIF-90-RhB in sections taken from light-lesioned retinas from 1 to 7 days post-lesion (dpl). The lesion sites are indicated by the dotted rectangles. **c** Quantitative analysis of the fluorescence area (ANOVA; **P < 0.01). Scale bars in **a**: 500 μm; **b**: 20 μm. *RPE* retinal pigment epithelium; *OSL* outer segment layer; *ONL* outer nuclear layer; *ON* optic nerve

The in vitro imaging capability of ZIF-90-RhB was determined in BV-2 cells. Red fluorescence signals were localized in the cytoplasm and nucleus in a dose- and time-dependent manner (Additional file 3: Fig. S3a–d; ANOVA, ***P < 0.001), indicating that ZIF-90-RhB successfully entered the cells and emitted red fluorescence. For the cell retention assessment, the fluorescence intensity showed a gradual declining trend, but homogeneous fluorescence signals were still observed at 5 days post-incubation (Additional file 3: Fig. S3e, f; ANOVA, *P < 0.05). The in vivo fluorescent labeling of ZIF-90-RhB was tracked with zebrafish embryos and larvae. The fluorescence signals were mainly distributed in the gastrointestinal tract at 96 and 120 h post-fertilization (hpf; Fig. 4a). To determine the distribution and retention time in the retina, ZIF-90-RhB was injected into the vitreous body of the zebrafish following light lesion, and cryosectioning was performed from 1 to 7 days post-lesion (dpl). Fluorescence signals were mainly detected at the lesion site until 7 dpl (Fig. 4b, dotted rectangles). The area of fluorescent signals increased steadily, peaked at 3 dpl, and decreased from 4 dpl (Fig. 4c; ANOVA, **P < 0.01), suggesting that ZIF-90-RhB remained in the retina for 7 days with only a single injection. These data indicate that ZIF-90-RhB has excellent imaging capability and photostability both in vitro and in vivo.

### Biocompatibility of ZIF-90-RhB

**Fig. 5:**
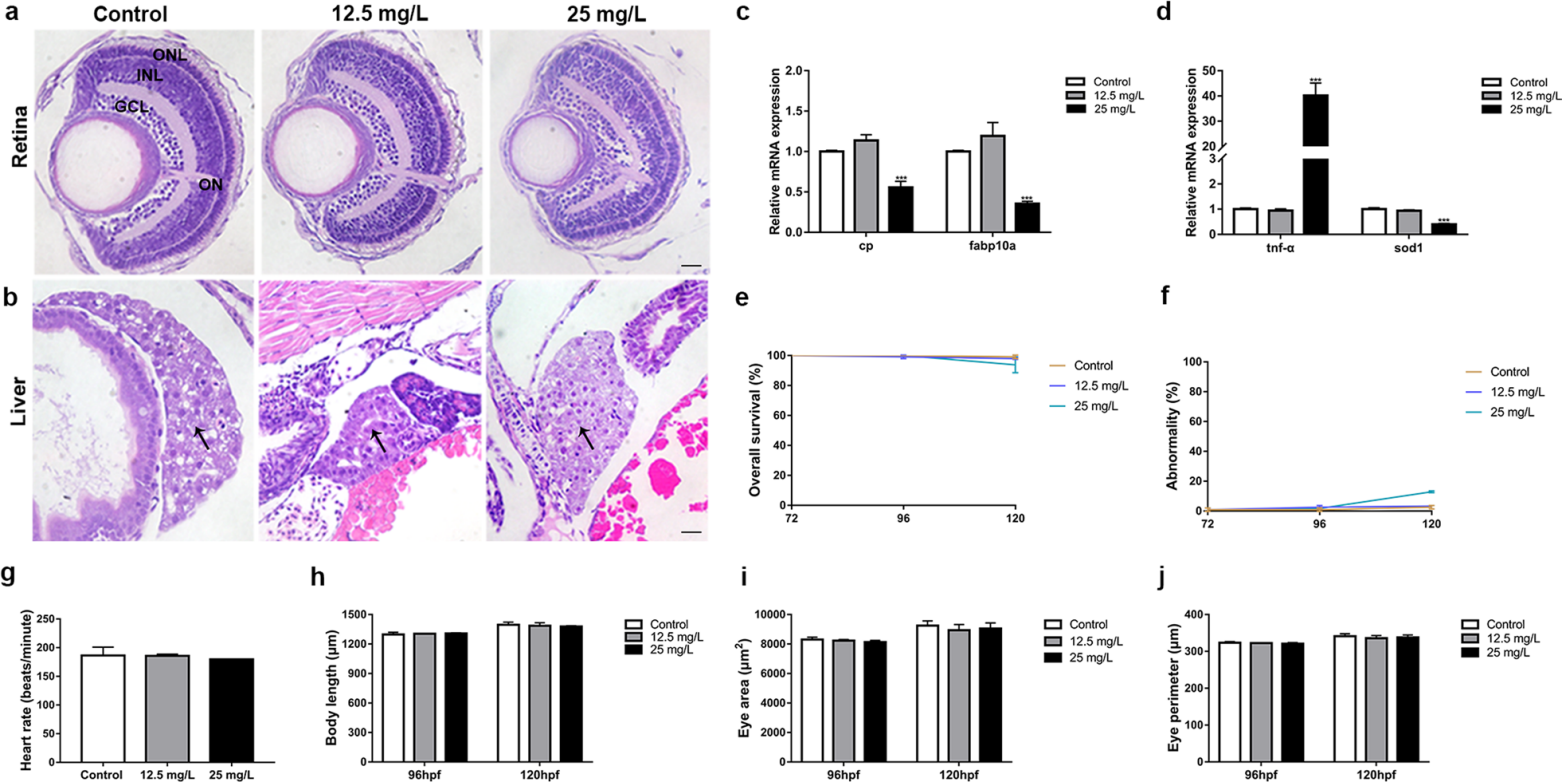
The biotoxicity of ZIF-90-RhB in larval zebrafish. **a** HE staining of the retina. **b** HE staining of the liver (arrows). **c** The relative expression levels of cp and fabp10a (ANOVA; ***P < 0.001). **d** The relative expression levels of tnf-α and sod1 (ANOVA; ***P < 0.001). **e**–**j** Statistical analysis of the **e** survival rate, **f** abnormality rate, **g** heart rate at 120 hpf, **h** body length, **i** eye area and **j** eye perimeter at 96 hpf and 120 hpf. No significant differences were found among the control, 12.5 mg/L- and 25 mg/L-exposed groups (ANOVA). Scale bars in **a**, **b**: 50 μm. *ONL* outer nuclear layer; *INL* inner nuclear layer; *GCL* ganglion cell layer; *ON* optic nerve

The in vitro cytotoxicity of ZIF-90-RhB was evaluated by MTT assay with BV-2 cells. No significant difference was found in cell viability at concentrations ranging from 3.125 to 50 mg/L (Additional file 4: Fig. S4a; ANOVA, *P* > 0.05). In vivo toxicity on retina and liver was carried out by HE staining in zebrafish larvae at 120 hpf. The retinas were well laminated among the control and ZIF-90-RhB-exposed groups at 12.5 mg/L- and 25 mg/L, respectively (Fig. 5a). In all three groups, the livers were composed of well-defined polygonal cells with abundant cytoplasm and prominent nuclei. No significant malformation was found in the hepatocyte and hepatic cord arrangement (Fig. 5b, arrows). Only the expression levels of the liver developmental markers ceruloplasmin (cp) and fabp10a were significantly decreased in the 25 mg/L group (see [26, 27]; Fig. 5c; ANOVA, ***P < 0.001). Quantitative real-time polymerase chain reaction (qRT-PCR) results showed that inflammatory cytokine tnf-α expression was significantly increased, while that of oxidative stress indicator sod1 was obviously decreased in the 25 mg/L group (Fig. 5d; ANOVA, ***P < 0.001). Moreover, no significant difference was found in survival rate, abnormality rate, heart rate, body length, eye area and eye perimeter among three groups (Fig. 5e–j; ANOVA, *P* > 0.05), indicating that ZIF-90-RhB exposure did not lead to abnormal development in zebrafish. Taken together, ZIF-90-RhB exposure at a concentration of 12.5 mg/L has low biotoxicity, both in vitro and in vivo.

### Microglial proliferation and inflammatory response following ZIF-90-RhB-GW2580 intraocular delivery

**Fig. 6:**
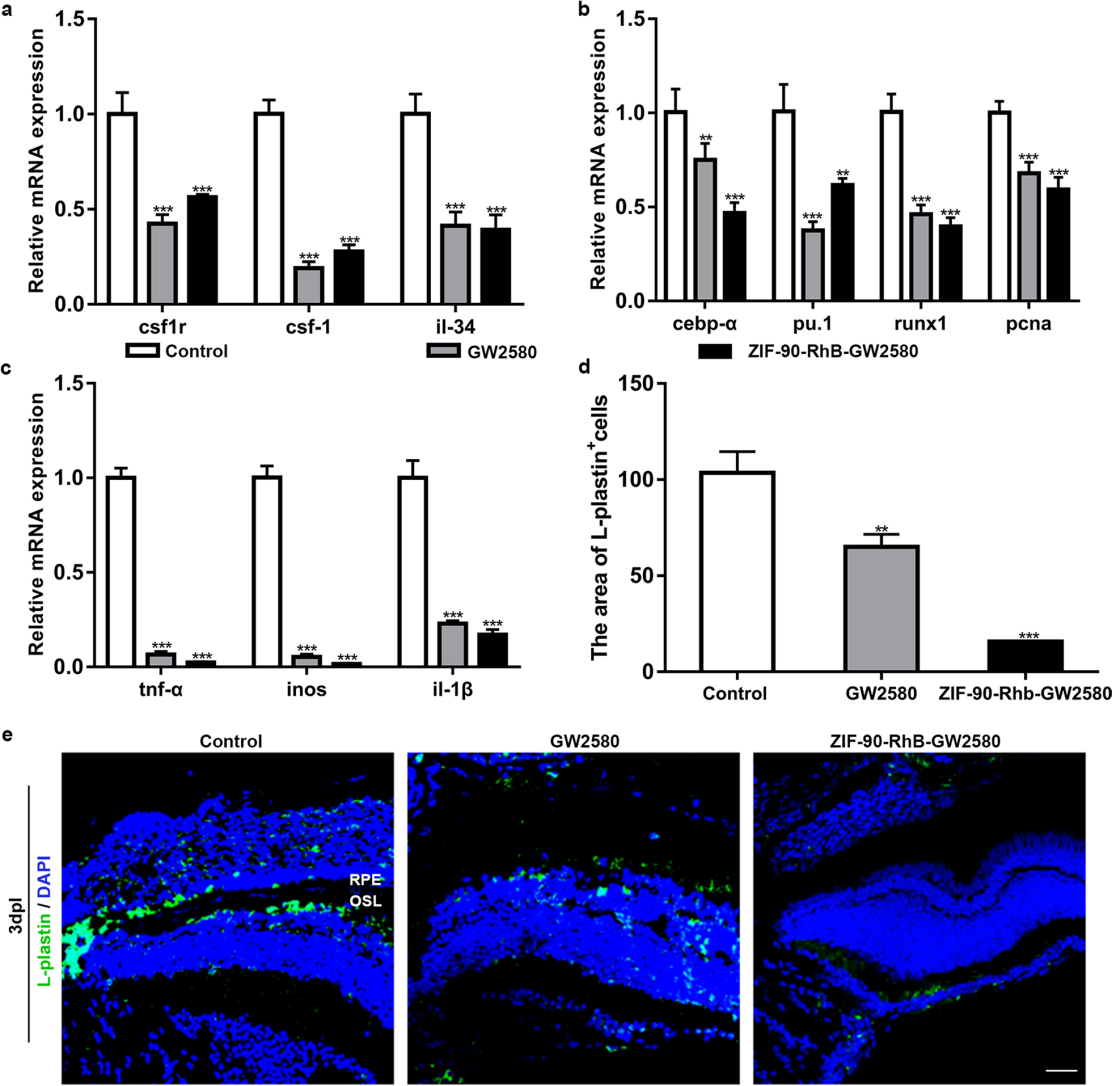
The inhibited microglial activation and the expression of inflammatory factors following ZIF-90-RhB-GW2580 treatment. **a** qRT‒PCR analysis of the expression levels of csf1r, csf-1 and il-34 in the control, GW2580 and ZIF-90-RhB-GW2580 groups at 3 days post-lesion (dpl) (ANOVA; ***P < 0.001). **b**, **c** The relative expression levels of **b** microglial proliferation-related markers (cebp-α, pu.1, runx1 and pcna) and **c** proinflammatory factors (tnf-α, inos and il-1β) at 3 dpl (ANOVA; **P < 0.01, ***P < 0.001). **d** Quantification of the fluorescence area of L-plastin-positive cells in sections taken from retinas in three groups at 3 dpl (ANOVA; **P < 0.01, ***P < 0.001). **e** Immunofluorescence images of L-plastin at 3 dpl. Scale bar in **e**: 20 μm. *RPE* retinal pigment epithelium; *OSL* outer segment layer

The MTT assay showed that the conjugation of ZIF-90-RhB with GW2580 did not increase the cytotoxicity (Additional file 4: Fig. S4b; ANOVA, *P* > 0.05). The prepared ZIF-90-RhB-GW2580 at a concentration of 12.5 mg/L was injected into the vitreous body and set as the ZIF-90-RhB-GW2580 group. Since the loading rate of GW2580 was approximately 8.2 wt%, the same volume of GW2580 at a concentration of 1.025 mg/L was injected and set as the GW2580 group, which ensured an equal dose of GW2580 between two groups. The expression of csf1r and its ligands (csf-1 and il-34) was measured by qRT-PCR in retinas. At 3 dpl, their expression levels in the control, GW2580 and ZIF-90-RhB-GW2580 groups declined significantly (Fig. 6a; ANOVA, ***P < 0.001). Meanwhile, the expression levels of microglial proliferation-related markers (cebp-α, pu.1, runx1 and pcna) were significantly downregulated in the GW2580 and ZIF-90-RhB-GW2580 group (Fig. 6b; ANOVA, **P < 0.01, ***P < 0.001). Similarly, the expression levels of the inflammatory factors tnf-α, inos and il-1β were markedly decreased (Fig. 6c; ANOVA, ***P < 0.001). Immunohistochemistry was performed with the L-plastin antibody to further depict the distribution of microglia [28]. In the control group, L-plastin-positive cells aggregated mainly around the lesion site in the outer segment layer and retinal pigment epithelium in an amoeboid shape with enlarged cell bodies; in contrast, few L-plastin-positive cells with smaller sizes were detected in the GW2580 and ZIF-90-RhB-GW2580 groups, especially in the ZIF-90-RhB-GW2580 group (Fig. 6d, e; ANOVA, **P < 0.01, ***P < 0.001). The above data not only clarify the effective inhibition of CSF1R in vivo, but also demonstrate that intraocular delivery of ZIF-90-RhB-GW2580 suppresses the microglial proliferation and inflammatory response significantly.

### Morphology, retinal structure and visual function following ZIF-90-RhB-GW2580 intraocular delivery

**Fig. 7:**
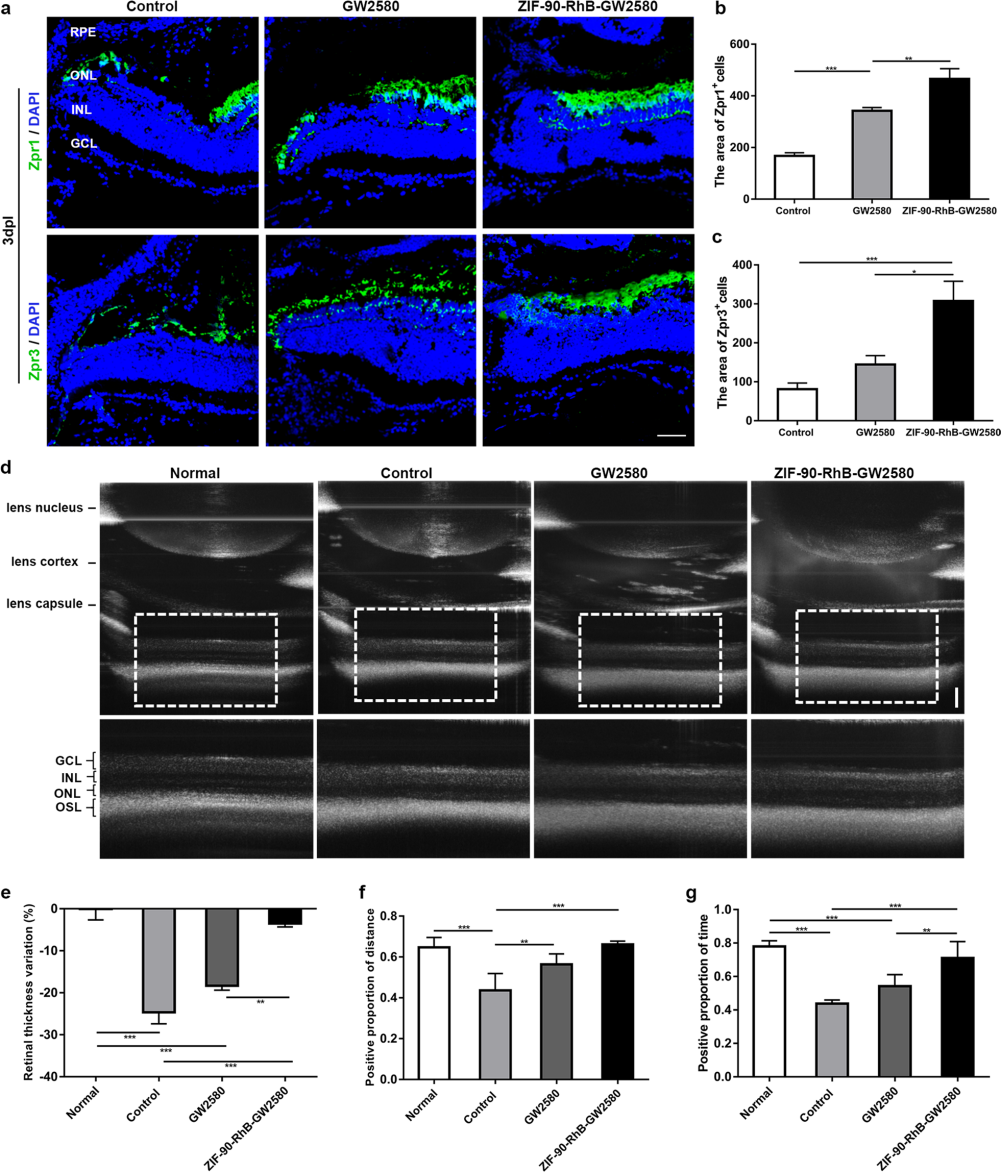
The expression of photoreceptor markers, retinal structure, and optomotor response following treatment with ZIF-90-RhB-GW2580. **a** Zpr1 and Zpr3 immunofluorescence staining in sections taken from retinas in the control, GW2580 and ZIF-90-RhB-GW2580 groups at 3 days post-lesion (dpl). **b**, **c** Quantification of the fluorescence areas of **b** Zpr1- and **c** Zpr3-positive cells (ANOVA; *P < 0.05, **P < 0.01, ***P < 0.001). **d** Images of PS-OCT in retinas from the normal (unlesioned and uninjected), control, GW2580 and ZIF-90-RhB-GW2580 groups at 4 dpl. **e** Quantitative analysis of the retinal thickness variation (ANOVA; **P < 0.01, ***P < 0.001). **f**, **g** The statistical analyses of the **f **positive proportion of distance and **g **positive proportion of time at 4 dpl (ANOVA; **P < 0.01, ***P < 0.001). Scale bars in **a**: 20 μm; **d**: 100 μm. *RPE*, retinal pigment epithelium; *ONL* outer nuclear layer; *INL* inner nuclear layer; *GCL* ganglion cell layer; *OSL* outer segment layer

The effects of GW2580 or ZIF-90-RhB-GW2580 intraocular delivery on photoreceptor were evaluated in three aspects: morphology, retinal structure and visual function. First, we examined the arrangement of cones and rods among the control, GW2580 and ZIF-90-RhB-GW2580 groups at 3 dpl by immunohistochemistry. Zpr1-expressing cells were obviously disorganized or absent at the lesion site in the control and GW2580 groups; however, there was a short defect range of Zpr1-expressing cells in the ZIF-90-RhB-GW2580 group. Similarly, Zpr3-expressing cells showed marked exfoliation in the control group, partial exfoliation in the GW2580 group, and slight disorganization in the ZIF-90-RhB-GW2580 group (Fig. 7a). Statistical analysis showed that the fluorescent areas of Zpr1- or Zpr3-expressing cells were increased following GW2580 injection, especially in the ZIF-90-RhB-GW2580 group (Fig. 7b, c; ANOVA, *P < 0.05, **P < 0.01, ***P < 0.001). Second, the retinal structure was determined by polarization sensitive optical coherence tomography (PS-OCT) at 4 dpl (Fig. 7d). Compared to the normal group, the relative thickness variation was significantly increased in the control and GW2580 groups and the minimum relative thickness variation was found in retinas from the ZIF-90-RhB-GW2580 group (Fig. 7e; ANOVA, **P < 0.01, ***P < 0.001). Interestingly, the relative thickness variation between the normal and ZIF-90-RhB-GW2580 groups was not statistically different. Finally, visual function assessment was performed using an optomotor response (OMR) behavioral test. The positive proportion of distance and the positive proportion of time in the control group were obviously lower than those in the normal group. In contrast, the two proportions were significantly increased in the GW2580 and ZIF-90-RhB-GW2580 groups (Fig. 7f, g; ANOVA, **P < 0.01, ***P < 0.001). Surprisingly, no significant difference was found between the normal and ZIF-90-RhB-GW2580 groups. Taken together, ZIF-90-RhB-GW2580 intraocular delivery alleviates the photoreceptor injury and remains almost normal retinal structure and visual function.

## Discussion

LPS stimulation was a classic approach to activate microglia in vitro [29]. The consequences of GW2580 treatment on BV-2 cells were determined based on two aspects, phenotypes and inflammatory response. Activated microglia are heterogeneous and can be divided into two opposite phenotypes: M1 and M2 [23]. M1 microglia initially respond to injury and infection, generally act as the first line to defend tissues and give rise to the destruction of invasive pathogens; in contrast, M2 microglia are closely related to tissue repair, extracellular matrix reconstruction and the alleviation of acute inflammation [30–32]. As a CSF1R inhibitor, GW2580 had a much higher affinity for CSF1R and did not deplete the microglial population completely which maintained the beneficial effects of microglia [33]. The in vitro GW2580 treatment promoted the phenotypic transformation of microglia from a proinflammatory M1 to an immunosuppressive M2 and relieved the inflammatory response. We constructed an in vitro coculture system composed of LPS-stimulated microglia and H_2_O_2_-induced photoreceptors to mimic pathological events during photoreceptor degeneration to some extent [34–36]. Apoptosis was considered the ultimate pathway for photoreceptor death, either in human retinas or animal models of photoreceptor degeneration [37, 38]. We found that the inhibition of microglia-mediated inflammatory response eventually led to a blockade of photoreceptor apoptosis. Hence, mastering the phase transition of the M1/M2 phenotype within an appropriate time window to suppress the inflammatory response could potentially prevent or slow down the photoreceptor death.

Although the above work found a protective effect of GW2580 on photoreceptors, the short in vivo half-life of GW2580 has been a major obstacle to medication. Pharmacokinetic experiments in mice found that GW2580 was undetectable at 12 h [39]. To improve its bioavailability, we designed and developed a ZIF-90-based delivery system and RhB was chosen as the fluorochrome. On the one hand, RhB can emit bright red fluorescence; on the other hand, its non-planar structure with a three-dimensional size matches the pore and window sizes of ZIF-90. Our strategy was to (1) prepare nanoscale ZIF-90-RhB and evaluate the toxicity and imaging ability both in vitro and in vivo; and (2) use ZIF-90-RhB as the skeleton and encapsulate GW2580 to synthesize novel nanoparticles ZIF-90-RhB-GW2580. Remarkably, the one-step self-assembly method was a rapid and versatile synthetic approach, circumventing the need for high temperatures and prolonged reaction times. In zebrafish larvae, the fluorescent signals distributed mainly in the gastrointestinal tract due to the swallowing and digestion. 120 hpf was selected as the time point to test the toxicity because most organs were fully differentiated and had started to function [40]. It was reported that 30–99% of administered nanoparticles accumulated and were sequestered in the liver [27, 41]; besides, inflammation and oxidative stress are common manifestations of toxicity following exposure to nanoparticles [42]. We found that the abnormal expression of cp, fabp10, tnf-α and sod1 was only detected at high dose. ZIF-90-RhB exposure did not lead to obvious malformation. Moreover, ZIF-90-RhB exposure at a low dose did not induce toxicity, inflammatory response or oxidative stress in zebrafish. Based on properties including nanoscale size, porous structure, excellent imaging capability and favorable biocompatibility, ZIF-90-RhB was an ideal carrier skeleton to load drugs.

In adult zebrafish, intense and short-term light exposure specifically killed cones and rods in a narrow band of the central retina, providing an excellent model of photoreceptor degeneration [3]. Without intervention, the apoptosis of cones and rods reached its peak at 3 dpl [43], so that we observed a massive cell defect in the control group. The structural and functional changes were evaluated by PS-OCT and OMR at 4dpl in this study. OCT is a noninvasive technique which is widely used in the diagnosis of retinal diseases. As a functional extension of OCT, our homemade PS-OCT platform has the advantages of no labeling, high resolution, high sensitivity and high penetration depth [44]. OMR reflects the instinct that zebrafish with normal vision would chase a rotating grating [45]. The photoreceptor ablation led to an obvious thinning in the central retina and decreased the visual acuity. Intravitreous injection is currently the most common procedure in the treatment of vitreoretinal diseases and minimizes the side effects of systemic administration [46]. With a single injection, ZIF-90-RhB was remained at the lesion site for 7 days. This long retention time ascribed to the porous structure. Meanwhile, it avoided the adverse outcomes of repeat injections. The intraocular delivery of GW2580 or ZIF-90-RhB-GW2580 optimized the drug administration which enabled the precise delivery to the lesion site. With GW2580 injection, the microglial proliferation was reduced and the inflammatory microenvironment was relieved. Due to sustained release, ZIF-90-RhB-GW2580 exhibited more efficient and durable inhibitive effects and enhanced the efficacy of GW2580. So, only minor cell ablation was observed following ZIF-90-RhB-GW2580 injection. Accordingly, the retinal structure and visual acuity were close to normal. Overall, we identified the neuroprotective potential of GW2580 on photoreceptors and found that the intraocular delivery of ZIF-90-RhB-GW2580 nanoparticles at the early stage can prevent the progression of photoreceptor degeneration.

## Conclusion

In summary, GW2580 protects photoreceptors from oxidative stress. The underlying mechanisms involve the inhibition of microglial activation and proliferation and the relief of inflammatory microenvironment. ZIF-90-RhB is an ideal skeleton for loading drug due to its excellent biocompatibility and low toxicity. At the early stage of light injury, a single intraocular injection of ZIF-90-RhB-GW2580 enables the precise delivery and sustained release of the GW2580, preventing the progression of photoreceptor degeneration more effectively. The ZIF-90-RhB-GW2580 intraocular delivery system provides a promising approach for the treatment of retinal degenerative diseases.

## Materials and methods

### Reagents

The following reagents were used to synthesize nanoparticles: imidazolate-2-carboxyaldehyde (ICA; Alfa Aesar, Shanghai, China), Zn(CH_3_COOH)_2_·2H_2_O (HEOWNS, Tianjin, China), rhodamine B (RhB; Macklin, Shanghai, China), dimethylformamide (DMF; Concord Reagent, Tianjin, China) and GW2580 (Selleck Chemicals, Houston, TX, USA). Reagents were of at least analytical grade and were used as purchased without further purification.

### Synthesis of ZIF-90-RhB and ZIF-90-RhB-GW2580

For the synthesis of ZIF-90-RhB, 2 mL DMF solution of Zn(CH_3_COOH)_2_·2H_2_O (0.05 M) was added to 2 mL DMF solution of ICA (0.2 M) with 2.5 mM RhB. After vigorous stirring for 5 min, 10 mL DMF was added to the reaction mixture to further stabilize the spheres for 10 min, followed by washing with ethanol three times until no significant fluorescence signal was detected in the supernatant, after which the spheres were dried in vacuum for 24 h. ZIF-90-RhB-GW2580 nanoparticles were synthesized through a one-step self-assembly approach. Briefly, each 2 mL DMF of Zn(CH_3_COOH)_2_·2H_2_O (0.05 M) and 2 mL DMF of ICA (0.2 M) containing 2.5 mM RhB and 5 mM GW2580 were mixed under vigorous stirring. The remaining steps were the same as for the preparation of ZIF-90-RhB.

### Characterization of ZIF-90-RhB and ZIF-90-RhB-GW2580

The morphology of ZIF-90-RhB and ZIF-90-RhB-GW2580 nanoparticles was recorded by TEM (Hitachi, Japan). The hydration particle size and zeta potential were detected by DLS (Malvern, UK). The steady-state fluorescence was performed on FL-4600 fluorescence spectrometer (Hitachi, Japan). The compositions of ZIF-90-RhB and ZIF-90-RhB-GW2580 were analyzed by FTIR spectroscopy (Nicolet iS20; Thermo Fisher Scientific, Waltham, MA, USA). TGA was performed on a TG-DTA8122 thermal analyzer (Rigaku, Japan) at a rate of 10 °C/min in air. N_2_ adsorption–desorption isotherms and the pore size distribution were measured with a Micromeritics ASAP 2460 (Macmillan; Atlanta, GA, USA). The pore-size distribution was calculated by the Barrett, Joyner and Halenda (BJH) method according to the density functional theory (DFT) program. The PXRD patterns were obtained by a SmartLab 9KW (Rigaku, Japan) using Cu-Kα radiation (λ = 1.5418 Å).

### Cell lines and animal

The murine microglial BV-2 cell line and the photoreceptor-derived 661W cell line were purchased from ATCC and Guangzhou Jennio Biotech, respectively. Cells were cultured in Dulbecco's modified Eagle’s medium (DMEM; Biological Industries (BI), Israel) containing 10% (v/v) fetal bovine serum (FBS; BI) and 1% penicillin–streptomycin (Thermo Fisher Scientific). All cells were incubated at 37 °C with 5% CO_2_. Adult wild-type zebrafish (Tübingen strain, 3–6 months old) were obtained from the Institute of Hydrobiology, Chinese Academy of Sciences, and raised in a standard fish facility with a 14/10-h light/dark cycle at 28.5 °C [47]). Embryos or larvae were incubated in E3 medium (5 mM NaCl, 0.17 mM KCl, 0.33 mM CaCl_2_, and 0.33 mM MgSO_4_, pH 7.2) and staged by hpf.

### Cell treatment

For microglial activation, BV-2 cells were stimulated with culture medium containing LPS (Sigma-Aldrich, St. Louis, MO, USA) at a concentration of 100 μg/mL for 24 h. The 661W cells were induced with hydrogen peroxide (H_2_O_2_; Thermo Fisher Scientific) at a concentration of 600 μM for 24 h to generate oxidative stress in photoreceptors. GW2580 treatment was carried out in BV-2 cells at a concentration of 2 μM for 1 h before LPS stimulation. A cell coculture system was established in a Transwell chamber with polyester membranes (pore size = 0.4 μm). BV-2 cells were placed in the upper compartment, while 661W cells were placed in the lower compartment.

### Quantitative real-time polymerase chain reaction (qRT-PCR)

Total RNA was extracted from cells, larvae or eye cups of adult zebrafish using TRIzol (Thermo Fisher Scientific) reagent according to the manufacturer's protocol. Reverse transcription was carried out using TransScript First-Strand cDNA Synthesis SuperMix (Yeasen; Shanghai, China). Real-time PCR was performed using TransStart Top Green qPCR SuperMix (Yeasen). The protocol was as follows: 30 s at 94 °C followed by 45 cycles of 5 s at 94 °C, 30 s at 60 °C, and 10 s at 72 °C. The relative expression of mRNA was calculated by the 2^−∆∆Ct^ method. The experiment was repeated 3 times for each gene. The gene-specific primer sequences were listed in Additional file 5: Table S1.

### Flow cytometry

BV-2 and 661W cocultured cells were divided into three groups: cells without any reagent or treatment were used as the control group; cells stimulated with LPS and H_2_O_2_ served as the LPS + H_2_O_2_ group; and cells treated with GW2580 and subsequently stimulated with LPS and H_2_O_2_ served as the GW2580 + LPS + H_2_O_2_ group. To prepare single-cell suspensions, cells from each group were dissociated in 0.25% trypsin solution, washed twice in prechilled phosphate buffered saline (PBS; 0.1 M, pH 7.4), suspended in 1X binding buffer, incubated with FITC and PI for 10 min, and brought up with PBS to 500 μL. An annexin V-FITC/propidium iodide (PI) apoptosis kit was supplied by Solarbio (Beijing, China). FITC- and PI-labeled cells were sorted with a FACS Aria III system (Becton Dickinson, Franklin Lakes, NJ, USA) with a Coherent Innova 70 laser at 488 nm and 635 nm at 4 °C, respectively. The above experiment was repeated three times.

### In vitro biological imaging

For the in vitro biological imaging test, BV-2 cells were seeded in a 24-well plate and cultured for 24 h. To evaluate the bioimaging ability at different concentrations, cells were incubated in 6.25, 12.5 or 25 mg/L ZIF-90-RhB for 24 h. The time-lapse bioimaging test was performed in BV-2 cells incubated with ZIF-90-RhB solution at a concentration of 12.5 mg/L for 6, 12 or 24 h. For assessment of cell retention, BV-2 cells were incubated with ZIF-90-RhB at 12.5 mg/L for 24 h, washed 3 times with PBS, cultured in medium, and observed every 24 h for 5 days. Images of fluorescence were captured using an FV 1000 confocal microscope (Olympus Corporation, Tokyo, Japan). Red fluorescence images of ZIF-90-RhB and blue fluorescence of the DAPI staining were observed by using 559 nm and 405 nm excitation, respectively.

### In vitro cytotoxicity assay

BV-2 cells were plated into 96-well plates at 4 × 10^3^ cells per well and incubated with 100 µL of culture medium containing ZIF-90-RhB (0, 3.125, 6.25, 12.5, 25 and 50 mg/L) or ZIF-90-RhB-GW2580 (0, 3.125, 6.25, 12.5, 25 and 50 mg/L) for 24 h. An MTT kit (Keygen Biotech; Jiangsu, China) was used to evaluate the in vitro cytotoxicity, and each sample was assayed in at least three parallel replicates. The survival rate was calculated based on the absorbance measured at 560 nm wavelength by using a microplate reader (NanoQuant, Tecan, Switzerland).

### Light lesion of the retina and intravitreal injection

Photoreceptor degeneration was induced in adult zebrafish by intense light exposure as we described previously [48]. Fish were anesthetized with 0.1% tricaine (Sigma-Aldrich), and intravitreal injection was carried out 30 min later. For the distribution assay of ZIF-90-RhB in the retina, both eyes were injected with 1 μL of ZIF-90-RhB at a concentration of 12.5 mg/L. For the GW2580 treatment experiment, fish were divided randomly into three groups: the control, GW2580 and ZIF-90-RhB-GW2580 groups. Both eyes were injected with 1 μL of PBS, GW2580 (1.025 mg/L) or ZIF-90-RhB-GW2580 (12.5 mg/L). Following the operation, fish were revived in the system water, returned to the fish facility and raised normally.

### In vivo distribution of ZIF-90-RhB

Embryos were placed in a 6-well plate (50 embryos/well) at 48 hpf and exposed to ZIF-90-RhB until 120 hpf at concentrations of 12.5 and 25 mg/L. The same number of zebrafish embryos was raised in E3 medium as the control group. Embryonic phenotypes and the fluorescence distribution of ZIF-90-RhB in tissues were imaged with an LSM710 confocal microscope (Carl Zeiss, Jena, Germany). The ZIF-90-RhB-injected fish were anesthetized with 0.1% tricaine and euthanized immediately. Cryosections taken from the eye cups at 1, 2, 3, 4, 5, 6 and 7 days post-lesion (dpl) were washed 3 times with PBS and counterstained with 4′6-diamidino-2-phenylindole (DAPI; Sigma-Aldrich). Five fish were tested at each time point.

### In vivo toxicity

Embryos at 48 hpf were exposed to 12.5 or 25 mg/L ZIF-90-RhB until 120 hpf. Phenotypes were recorded with a DP72 digital camera mounted on an SZX16 stereomicroscope (Olympus) at 96 and 120 hpf, respectively. The overall survival rate, abnormality rate, heart rate, body length, eye area and eye perimeter were calculated to evaluate whether ZIF-90-RhB would affect zebrafish development.

### Immunofluorescence and immunohistochemistry

For cell immunofluorescence staining, BV-2 cells or 661W cells were seeded into a 24-well plate at a density of 5 × 10^4^/mL in 500 µL/well for growth on glass coverslips. Cells were washed 3 times with PBS containing 0.5% Triton X-100 for 5 min, fixed in 4% paraformaldehyde (PFA) for 20 min, and then stained. The primary antibodies were iNOS (Cell Signaling; Danvers, MA, USA), CD206 (Cell Signaling), Opn1mw (Millipore; Billerica, MA, USA) and NeuN (Millipore). The secondary antibody was a Cy3-conjugated antibody (Millipore). For immunohistochemistry, ZIF-90-RhB-exposed larvae at 120 hpf or injected adult fish were anesthetized with 0.1% tricaine and euthanized immediately. At selected time points, larvae or eye cups from adult fish were fixed in 4% PFA overnight at 4 °C, dehydrated in 20% sucrose in PBS for 1 h at room temperature, embedded in optimal cutting temperature compound (Sakura Finetek; Torrance, CA, USA) and processed for cryosectioning at 10 μm. The primary antibodies were Zpr1 [Zebrafish International Resource Center (ZIRC); Eugene, OR, USA], Zpr3 (ZIRC) and L-plastin (GeneTex; San Antonio, CA, USA), which were used to label cones, rods, ganglion cells and microglia, respectively. The secondary antibody was an Alexa Fluor 488 antibody (Abcam; Cambridge, MA, USA). DAPI was used to label the nuclei. Images of immunostaining were captured using an FV1000 confocal microscope (Olympus).

### Hematoxylin and eosin (HE) staining

Larvae at 120 hpf were anesthetized with 0.1% tricaine, euthanized immediately, fixed in 4% PFA overnight at 4 °C and embedded in paraffin. Sagittal slices were then prepared at a thickness of 5 µm. HE staining was performed using standard protocols [49]. Images of HE staining were captured with a BX51 microscope (Olympus).

### Polarization sensitive optical coherence tomography (PS-OCT)

The unlesioned and uninjected fish were defined as the normal group to determine the baseline. Fish from the normal, control, GW2580 and ZIF-90-RhB-GW2580 groups were anesthetized with 0.1% tricaine at 4 dpl. The pupils were dilated using tropicamide (Tropicil Top®; Alcon Laboratories, Texas, USA). Oxybuprocaine (Anestocil®; Théa Pharma, Schaffhausen, Switzerland) was applied to the cornea for topical anesthesia. Carmellose sodium (Celluvisc®; Sigma-Aldrich) was used to prevent corneal dryness. The retinal structure was scanned and photographed with a homemade PS-OCT system [44], and the thickness (from ganglion cell layer to outer segment layer) was calculated. Eight fish were scanned in each group. The relative thickness variation was determined with the equation:

$${\text{Relative}}\,{\text{thickness}}\,{\text{variation}}\, = \,\frac{X - Xi}{X}\, \times \,100\%$$

*X* represents the retinal thickness from the control, GW2580 or ZIF-90-RhB-GW2580 groups;* Xi*represents the retinal thickness from normal group (base line).

### Behavioral test

All behavioral tests were carried out at 14:00–18:00 to avoid the potential influence of circadian rhythms with an optomotor response (OMR) apparatus as we described previously [48]. The temporal and spatial frequencies of OMR were set at 30 rotations/minute (rpm) and 0.03 cycles/degree (c/deg), respectively. The OMR test was performed on fish from the normal, control, GW2580 and ZIF-90-RhB-GW2580 groups (n = 8 in each group) at 4 dpl. All digital tracks were analyzed by Ethovision XT software (Noldus Information Technology, Wageningen, the Netherlands). The positive distance and time were defined as traveling along the grating direction. Two parameters, positive proportion of distance (distance of positive response/total distance) and positive proportion of time (time of positive response/total time), were used to quantify the OMR performance.

### Statistical analysis

For image analysis, ImageJ software (1.8.0X, NIH, http://rsb.info.nih.gov/ij/) was used to convert the fluorescent images into 8-bit grayscale images prior to threshold processing and subsequently calculate the positive areas or intensity. Statistical analysis was performed with Prism software (version 9.0, GraphPad Software, La Jolla, USA). The values are shown as the mean ± standard deviation (SD) or standard error of the mean (SEM). *P* < 0.05 was considered statistically significant. Values from more than two groups were analyzed using one-way analysis of variance (ANOVA).

## Supplementary Information

**Additional file 1: Figure S1.** The expression levels of csf1r and its cognate ligands in LPS-stimulated BV-2 cells. (a-c) The relative expression levels of (a) csf1r and its cognate ligands (b) csf-1 and (c) il-34 in LPS-stimulated BV-2 cells at 0 (control), 6, 12, 24 and 48 hours (ANOVA; ***P<0.001).

**Additional file 2: Figure S2.** Fourier transform infrared (FTIR) spectra of ZIF-90-RhB and ZIF-90-RhB-GW2580. The peaks at 3150 cm^-1^ (arrow) and 1675 cm^-^^1^ (dotted line) are ascribed to the characteristic absorption of the benzene ring in the GW2580 and the stretching vibration of the aldehyde group in the ICA ligand, respectively.

**Additional file 3: Figure S3.** *In vitro* fluorescence imaging of ZIF-90-RhB. (a) Fluorescence images of BV-2 cells following ZIF-90-RhB exposure for 24 hours at different concentrations (0, 6.25, 12.5 and 25 mg/L). (b) Fluorescence images of BV-2 cells following ZIF-90-RhB exposure at 0, 6, 12 and 24 hours. (c and d) Statistical analysis of the fluorescence intensities of (a) and (b), respectively (ANOVA; ***P<0.001). (e) Time-lapse fluorescence images of BV-2 cells following 24-hour ZIF-90-RhB incubation from 1 to 5 days. (f) Quantitative analysis of the mean fluorescence intensity from (e) (ANOVA, *P<0.05). dpi, day (s) post incubation. Scale bars in (a), (b) and (e): 20 μm.

**Additional file 4: Figure S4.** MTT assay of ZIF-90-RhB and ZIF-90-RhB-GW2580. (a and b) The cell viabilities of (a) ZIF-90-RhB and (b) ZIF-90-RhB-GW2580 at different concentrations from 3.125 to 50 mg/L.

**Additional file 5: Table S1.** Primer sequences for qRT-PCR.

## Data Availability

All data generated or analysed during this study are included in this published article and its Additional files.

## Abbreviations

DAPI: 4′6-Diamidino-2-phenylindole
dpl: Days post lesion
DMF: Dimethylformamide
DMEM: Dulbecco’s modified Eagle’s medium
DLS: Dynamic light scattering
FBS: Fetal bovine serum
FTIR: Fourier transform infrared
HE: Hematoxylin and eosin
hpf: Hours post fertilization
H_2_O_2_: Hydrogen peroxide
ICA: Imidazolate-2-carboxyaldehyde
OMR: Optomotor response
PS-OCT: Polarization-sensitive optical coherence tomography
qRT-PCR: Quantitative real-time polymerase chain reaction
RhB: Rhodamine B
TGA: Thermogravimetric analysis
TEM: Transmission electron microscopy
PXRD: Powder X-ray diffraction
ZIF-90: Zeolitic imidazolate framework-90
