# Supplementary figures and images for: Intraocular delivery of ZIF-90-RhB-GW2580 nanoparticles prevents the progression of photoreceptor degeneration
Source: J Nanobiotechnology. 2023 Feb 6;21:44. doi: 10.1186/s12951-023-01794-6 (PMC9901128; doi:10.1186/s12951-023-01794-6)

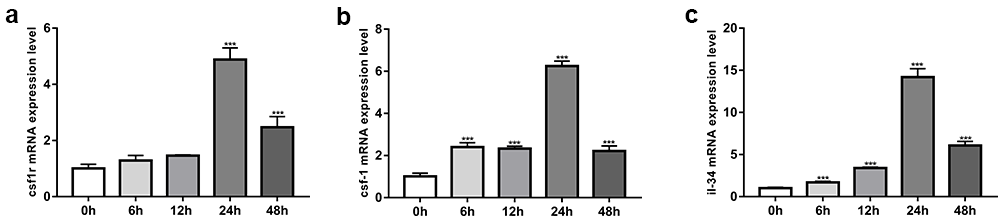

Supplement: Supplementary file 1 — Additional file 1: Figure S1. The expression levels of csf1r and its cognate ligands in LPS-stimulated BV-2 cells. (a-c) The relative expression levels of (a) csf1r and its cognate ligands (b) csf-1 and (c) il-34 in LPS-stimulated BV-2 cells at 0 (control), 6, 12, 24 and 48 hours (ANOVA; ***P<0.001). [file 12951_2023_1794_MOESM1_ESM.tif]

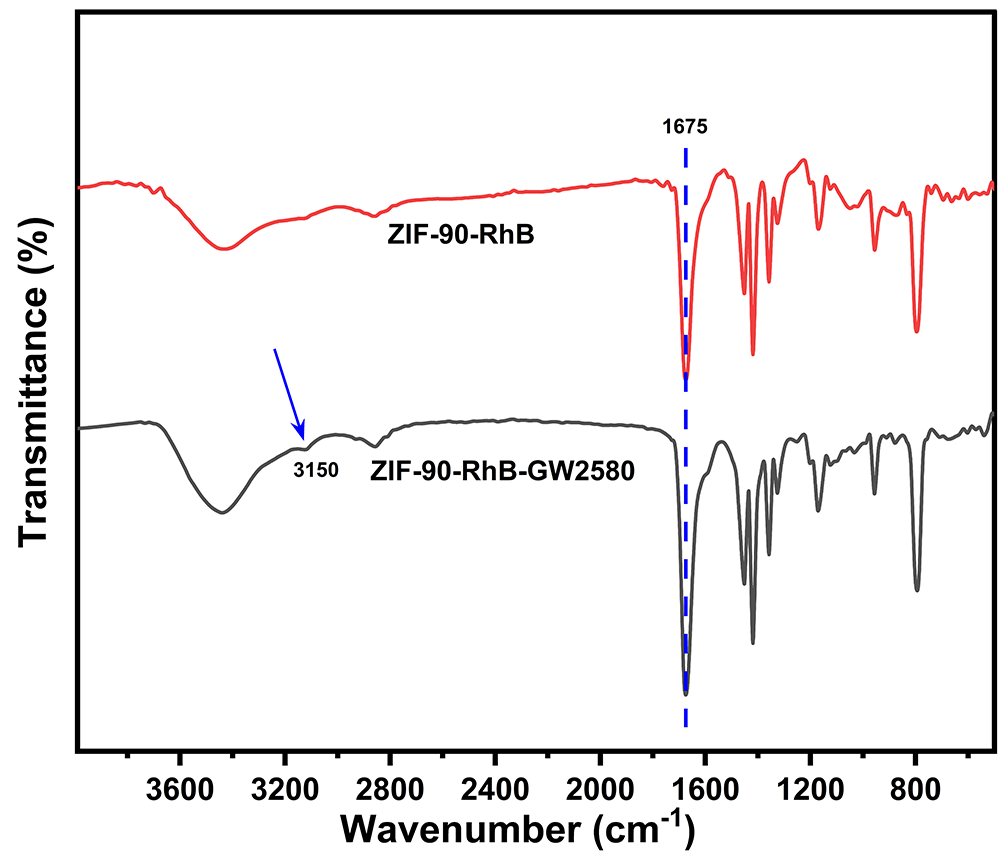

Supplement: Supplementary file 2 — Additional file 2: Figure S2. Fourier transform infrared (FTIR) spectra of ZIF-90-RhB and ZIF-90-RhB-GW2580. The peaks at 3150 cm-1 (arrow) and 1675 cm-1 (dotted line) are ascribed to the characteristic absorption of the benzene ring in the GW2580 and the stretching vibration of the aldehyde group in the ICA ligand, respectively. [file 12951_2023_1794_MOESM2_ESM.tif]

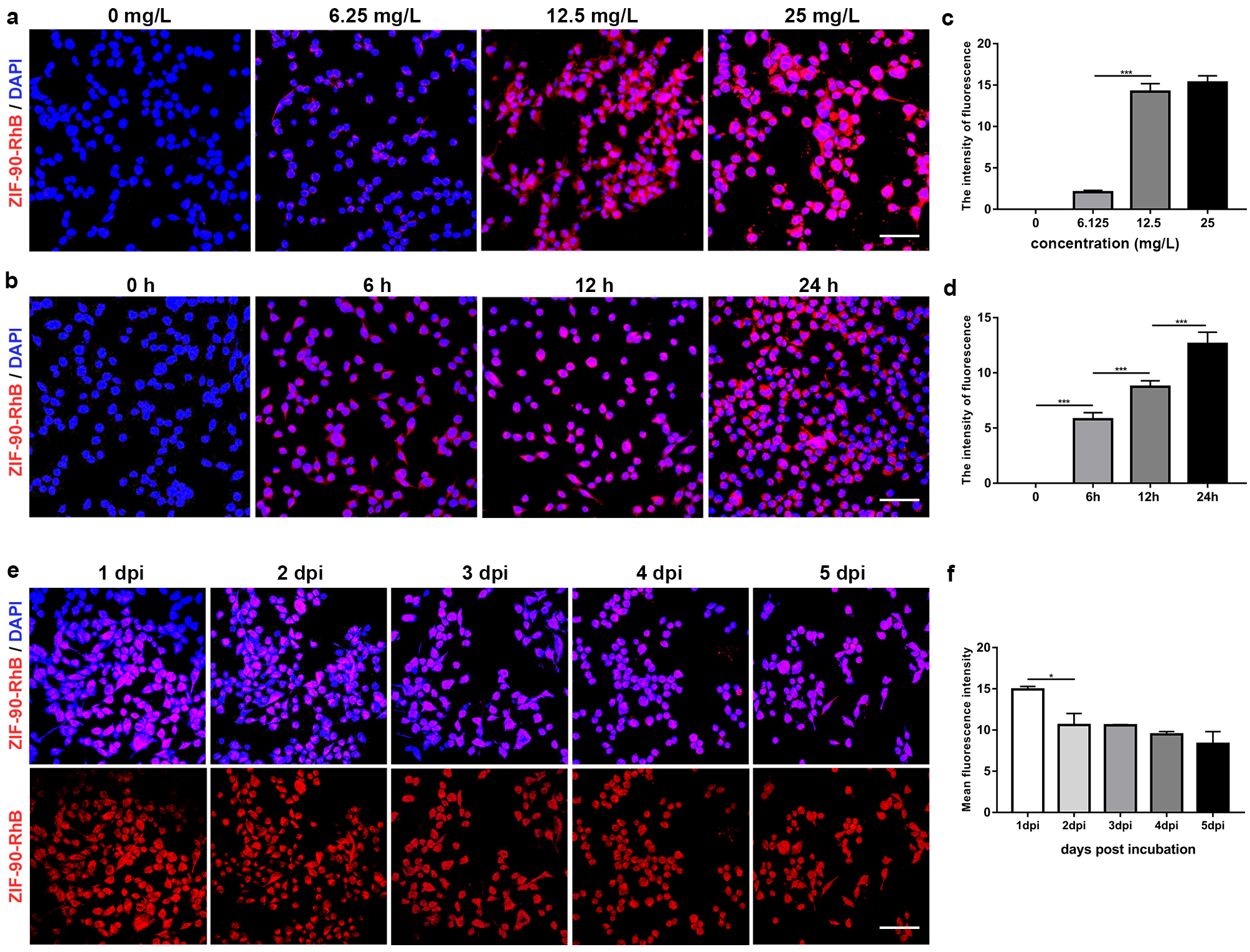

Supplement: Supplementary file 3 — Additional file 3: Figure S3. In vitro fluorescence imaging of ZIF-90-RhB. (a) Fluorescence images of BV-2 cells following ZIF-90-RhB exposure for 24 hours at different concentrations (0, 6.25, 12.5 and 25 mg/L). (b) Fluorescence images of BV-2 cells following ZIF-90-RhB exposure at 0, 6, 12 and 24 hours. (c and d) Statistical analysis of the fluorescence intensities of (a) and (b), respectively (ANOVA; ***P<0.001). (e) Time-lapse fluorescence images of BV-2 cells following 24-hour ZIF-90-RhB incubation from 1 to 5 days. (f) Quantitative analysis of the mean fluorescence intensity from (e) (ANOVA, *P<0.05). dpi, day (s) post incubation. Scale bars in (a), (b) and (e): 20 μm. [file 12951_2023_1794_MOESM3_ESM.tif]

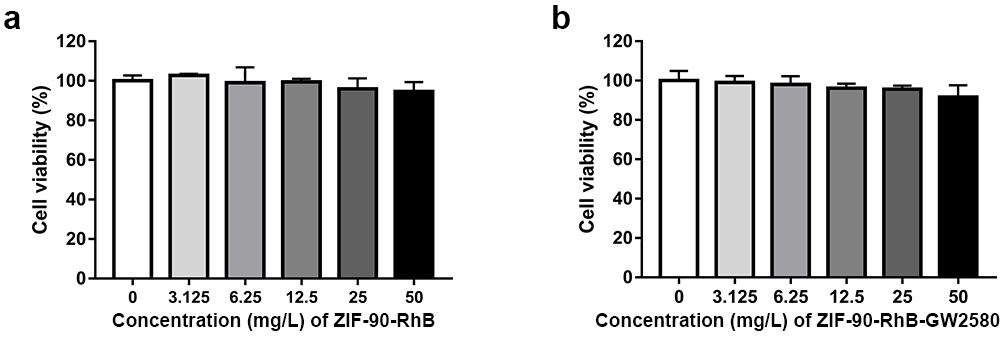

Supplement: Supplementary file 4 — Additional file 4: Figure S4. MTT assay of ZIF-90-RhB and ZIF-90-RhB-GW2580. (a and b) The cell viabilities of (a) ZIF-90-RhB and (b) ZIF-90-RhB-GW2580 at different concentrations from 3.125 to 50 mg/L. [file 12951_2023_1794_MOESM4_ESM.tif]
