# Supplementary material for: Intraocular delivery of ZIF-90-RhB-GW2580 nanoparticles prevents the progression of photoreceptor degeneration
Source: J Nanobiotechnology. 2023 Feb 6;21:44. doi: 10.1186/s12951-023-01794-6 (PMC9901128; doi:10.1186/s12951-023-01794-6)
Supplement: Supplementary file 5 — Additional file 5: Table S1. Primer sequences for qRT-PCR. [file 12951_2023_1794_MOESM5_ESM.docx]

**Table S1. Primer sequences for qRT-PCR.**

| Gene | GenBank | Sequence |
| --- | --- | --- |
| β-actin | NM_131031 | Forward: 5’-CACCACAGCCGAAAGAG-3’  Reverse: 5’-GATACCGCAAGATTCCATAC-3’ |
| cebp-α | NM_131885 | Forward: 5’-GCCTACATTGATCCGTCTGCCTTC-3’  Reverse: 5’-CCGTGGTGGTAGTCGTAGTCTCC-3’ |
| cp | NM_131802 | Forward: 5’-GGCAGGAGATAGACAAACC-3’  Reverse: 5’-TGCGTGGAGGGAGTAAG-3’ |
| csf-1 | NM_001114480 | Forward: 5’-GGTGTGCCAGGTCCATGTAAGC-3’  Reverse: 5’-TCTGACGCTCTGTGAAGGTGTAGG-3’ |
| csf1r | NM_131672 | Forward: 5’-CCGCCTGCCTGTAAAGTGGATG-3’  Reverse: 5’-CCAGAGGAGGATGCCGTAGGAC-3’ |
| fabp10a | NM_152960 | Forward: 5’-ATGGACGGCAAGAAGCTCAAGTG-3’  Reverse: 5’-TGGTGGTTCCTCCGACTGTCAG-3’ |
| il-1β | NM_212844 | Forward: 5’-TTCCCCAAGTGCTGCTTATT-3’  Reverse: 5’-AAGTTAAAACCGCTGTGGTCA-3’ |
| il-34 | NM_001128701 | Forward: 5’-GAGGAGGTGTTCAGACTGCGAAAC-3’  Reverse: 5’-ATGCCCTGTTGACTCACGTAAAGC-3’ |
| inos | NM_001104937 | Forward: 5’-TTTGGCCTGGGTTCACGT-3’  Reverse: 5’-TCGTCCTGCCCGTTTAGTT-3’ |
| pcna | NM_131404 | Forward: 5’-CGACAAGGAGGATGAAGCGGTAAC-3’  Reverse: 5’-GACAGAGGAGTGGCTTTGGTGAAG-3’ |
| pu.1 | NM_001328368 | Forward: 5’-TGCTTGACCTTCTGCGAAATGGAG-3’  Reverse: 5’-TTGCGATTGCCCTTCTGGATGC-3’ |
| runx1 | NM_131603 | Forward: 5’-ACAGTGATGGCGGGAAATGATGAG-3’  Reverse: 5’-GACGGTGATGGTGAGAGTGAAGC-3’ |
| sod1 | NM_131294 | Forward: 5’-ATCAAGAGGGTGAAAAGAAGC-3’  Reverse: 5’-AAAGCATGGACGTGGAAAC-3’ |
| tnf-α | NM_212859 | Forward: 5’-GAACAACCCAGCAAACTC-3’  Reverse: 5’-CATCACCAGCGGTAAAGG-3’ |
